# CNT/High Mass Loading MnO_2_/Graphene-Grafted Carbon Cloth Electrodes for High-Energy Asymmetric Supercapacitors

**DOI:** 10.1007/s40820-019-0316-7

**Published:** 2019-10-17

**Authors:** Lulu Lyu, Kwang-dong Seong, Jong Min Kim, Wang Zhang, Xuanzhen Jin, Dae Kyom Kim, Youngmoo Jeon, Jeongmin Kang, Yuanzhe Piao

**Affiliations:** 1 Graduate School of Convergence Science and Technology, Seoul National University, Seoul, 151-742 Republic of Korea; 2 School of Chemistry and Chemical Engineering, Yangzhou University, Yangzhou, 225002 Jiangsu People’s Republic of China; 3 Advanced Institutes of Convergence Technology, Suwon, 443-270 Republic of Korea

**Keywords:** High mass loading, Flexible pseudocapacitor, Voltage window, Energy density

## Abstract

Flexible supercapacitor electrodes with high mass loading are crucial for obtaining favorable electrochemical performance but still challenging due to sluggish electron and ion transport. Herein, rationally designed CNT/MnO_2_/graphene-grafted carbon cloth electrodes are prepared by a “graft-deposit-coat” strategy. Due to the large surface area and good conductivity, graphene grafted on carbon cloth offers additional surface areas for the uniform deposition of MnO_2_ (9.1 mg cm^−2^) and facilitates charge transfer. Meanwhile, the nanostructured MnO_2_ provides abundant electroactive sites and short ion transport distance, and CNT coated on MnO_2_ acts as interconnected conductive “highways” to accelerate the electron transport, significantly improving redox reaction kinetics. Benefiting from high mass loading of electroactive materials, favorable conductivity, and a porous structure, the electrode achieves large areal capacitances without compromising rate capability. The assembled asymmetric supercapacitor demonstrates a wide working voltage (2.2 V) and high energy density of 10.18 mWh cm^−3^.

**Electronic supplementary material:**

The online version of this article (10.1007/s40820-019-0316-7) contains supplementary material, which is available to authorized users.

## Highlights

- CNT/MnO_2_/graphene-grafted carbon cloth electrode is designed and achieves high MnO_2_ mass loading (9.1 mg cm^−2^).
- The electrode with favorable electronic/ionic conductivity delivers a large areal capacitance and rate capability.
- The assembled asymmetric supercapacitor yields a large energy density of 10.18 mWh cm^−3^.

**Electronic supplementary material:**

The online version of this article (10.1007/s40820-019-0316-7) contains supplementary material, which is available to authorized users.

## Graphical abstract

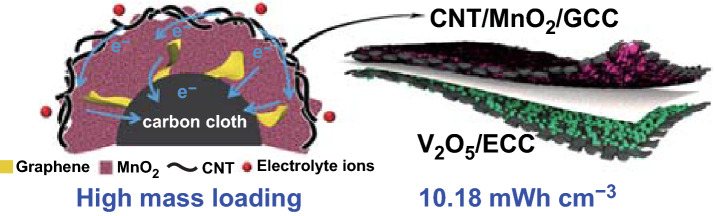

## Introduction

Flexible supercapacitors are one of the promising energy storage devices for wearable electronics due to their quick charging/discharging rate, high power density, and long cycle life [1]. However, it is challenging to store a large amount of energy in a confined device area, and further improvement would be necessary for practical applications. According to the equation of *E* = 0.5 *CV*^2^, the energy density (*E*) can be improved either by increasing the capacitance (*C*) or widening cell voltage (*V*). Hence, the combination of two electrodes with high capacitances and opposite potential windows to assemble asymmetric supercapacitor (ASC) is a feasible method to obtain a high energy density.

Pseudocapacitive materials are normally deposited on a flexible scaffold to obtain a high energy density. In particular, manganese dioxide (MnO_2_) features a large theoretical capacitance, low cost, and environmental benignity [2–4]. The high specific capacitance that has been reported so far can be explained by the low mass loading (< 1 mg cm^−2^), while real-world applications require high mass loading around 10 mg cm^−2^ to obtain a high areal capacitance and energy density [5, 6]. However, due to poor electrical conductivity (10^−5^–10^−6^ S cm^−1^) and elongated electron/ion transport distances, high mass loading of MnO_2_ results in low electroactive areas, large resistance, along with sluggish mass diffusion and ion transport, severely limiting its energy storage capability [7]. Moreover, high mass loading often leads to easy detachment from flexible substrates because of the weak interfacial combination [7]. Therefore, it is still difficult to fabricate flexible electrodes that display high mass loading, high areal capacitance and energy density, and structural stability.

One strategy to address the aforementioned issue is to modulate the crystalline structure of MnO_2_ through electrochemical method [7], metal atom doping [8, 9], or Ostwald ripening [10], which increases the conductivity and electroactive sites. Another strategy is to deposit MnO_2_ on a conductive and high-surface-area scaffold to augment its capacitive utilization [11, 12]. Considering the limited surface area of flexible substrates like Ni foam or carbon cloth (CC), secondary conductive templates are built on them to provide additional space that allows high MnO_2_ mass loading [13]. Meanwhile, secondary materials as ideal conductive “bridges” can increase the electrical contact area between active species and current collectors, which effectively guarantees their strong adhesion, reduces the interfacial resistance, and enhances the transport efficiency of electrons and ions [14]. As such, Liu et al. [15] showed that the deposition of the metal–organic framework-based carbon on CC can increase the surface area of the substrate and allow for high mass loading of electroactive materials, more ion-accessible spaces, and effective electron transport, contributing to a high capacitance and fast power delivery. Likewise, Yang’s group [16] coated carbon nanoparticles on carbon fibers to supply sufficient sites for high MnO_2_ mass loading and facilitate electron/ion transport, beneficial for high capacitive utilization of pseudocapacitive materials. More importantly, to maintain the capacitances of high mass loading at large current densities, charge transfer at the electrolyte/electrode interface also should be optimized to further augment reaction kinetics, ensuring a highly reversible charging/discharging process [17, 18]. Accordingly, a rational design for electrodes with high mass loading should include a three-dimensional (3D) porous structure for rapid mass diffusion, high conductivity for efficient electron transport, and sufficient ion-accessible areas for redox reactions.

**Scheme 1:**
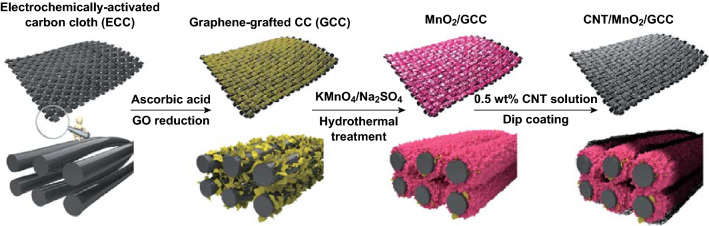
Schematic illustration of the preparation procedure of the CNT/MnO_2_/GCC electrode

Herein, we aim to enhance the areal capacitance and rate performance of flexible supercapacitor electrodes with high MnO_2_ mass loading by rationally designing the electrode architecture, as illustrated in Scheme 1. The high electrochemical performance of the 3D hierarchical CNT/MnO_2_/graphene-grafted CC (GCC) electrode can be explained by the following factors: (1) a high-surface-area GCC substrate for high MnO_2_ mass loading (9.1 mg cm^−2^) and electron transport; (2) uniform deposition of MnO_2_ on GCC favorable for structural stability; (3) interconnected CNT on MnO_2_ for rapid charge transfer; (4) nanostructured MnO_2_ with abundant electroactive sites and short ion transport pathways; (5) the absence of binders and additives. The electrode with high mass loading delivers an outstanding areal capacitance (3.38 F cm^−2^ at a current density of 1 mA cm^−2^) without sacrificing rate capability (53.3% capacitance retention from 1 to 30 mA cm^−2^). More importantly, the quasi-solid-state flexible ASC with CNT/MnO_2_/GCC cathode and V_2_O_5_ anode shows a wide working voltage (2.2 V), a large energy density (10.18 mWh cm^−3^), and good cycling stability.

## Experimental Section

### Materials

Carbon cloth was obtained from NARA Cell-Tech Corporation. Ammonium sulfate ((NH_4_)_2_SO_4_), ascorbic acid, and potassium persulfate were obtained from Samchun Chemicals. Other chemicals were purchased from Sigma-Aldrich.

### Preparation of Samples

#### Preparation of GCC

CC was electrochemically activated in 0.1 mol (NH_4_)_2_SO_4_ solution under an applied voltage of 10 V for 10 min to improve its wettability [19]. The surface of electrochemically activated CC (ECC) was modified with positive charges by immersing it in a 20 mL 0.5 wt% poly(diallyldimethylammonium chloride) (PDDA) aqueous solution containing 20 mmol NaCl and 20 mmol Tris [20]. The mixture was stirred at room temperature for 2 h and then washed with deionized water. Thereafter, positively charged ECC was immersed into 2 mg mL^−1^ of negatively charged graphene oxide (GO) solution and made to stand for 1 h, during which GO nanosheets tended to anchor on carbon fibers because of the electrostatic interaction [21]. Ascorbic acid (40 mg) was added to the above solution followed by heating at 90 °C for 2 h. GO was reduced to graphene hydrogel that was covered on the entire ECC. Finally, GCC was prepared after washing residual graphene away. The preparation process of GO and CNT solution is described in Supporting Information.

#### Preparation of CNT/MnO_2_/GCC

GCC was immersed in a 10 mL solution containing 25 mmol KMnO_4_ and 25 mmol Na_2_SO_4_. The reaction medium was transferred to a Teflon-lined stainless steel autoclave followed by hydrothermal treatment at 150 °C for 3 h. The mass loading of MnO_2_ was adjusted by tuning the concentration of KMnO_4_ (20, 25, and 30 mmol). After cooling down to room temperature, MnO_2_/GCC was washed with deionized water three times and dipped into CNT solution (0.5 wt%) for 60 s. Finally, CNT/MnO_2−x_/GCC electrodes were dried in air. X in MnO_2−x_ represents the concentration of KMnO_4_ (20, 25, and 30 mmol) in the following text. CNT/MnO_2_/ECC electrodes were prepared with the same procedure but using bare ECC as the substrate.

#### Preparation of V_2_O_5_/ECC

V_2_O_5_/ECC was prepared by electrodepositing V_2_O_5_ on ECC (1 × 1 cm^2^) at a constant potential of 0.7 V in an aqueous solution containing 0.5 mol vanadyl sulfate and 0.5 mol ammonium acetate for 80 min. A saturated calomel electrode (SCE) and Pt wire were used as a reference and counter electrode, respectively. Then, V_2_O_5_/ECC was washed and dried at 60 °C under vacuum condition overnight. The mass loading of V_2_O_5_ was measured to be 11.8 mg cm^−2^.

### Characterizations

The morphology, crystal structure, and composition of samples were examined by a field emission scanning electron microscopy (FESEM, Hitachi S-4800), transmission electron microscopy (TEM), and high-resolution transmission electron microscopy (HRTEM, CM300 UT/FEG) with energy-dispersive X-ray (EDX) spectrometry, X-ray diffraction (XRD, Bruker D8 Advance with Cu kα radiation λ = 0.15406 nm), X-ray photoelectron spectroscopy (XPS, a PHI 5700 ESCA with Al kα radiation), and Raman spectrometer (LabRAM HR Evolution with a 514 nm laser beam). The specific surface area was calculated by a Brunauer–Emmett–Teller (BET) method. N_2_ adsorption/desorption isotherm was obtained from BELSORP-mini II. The specimen for TEM analysis was obtained by scratching active species from carbon cloth by tweezers. The mass loading of active materials was measured by an Ohaus DV215CD semi-microbalance.

### Electrochemical Measurements

Cyclic voltammetry (CV), galvanostatic charge–discharge (GCD), and electrochemical impedance spectroscopy (EIS) tests of electrodes were conducted on a CHI 660D electrochemical workstation in 1 M Na_2_SO_4_ aqueous electrolyte under a three-electrode configuration. The tested area of electrodes was fixed as 1 cm^2^. SCE and Pt wire were used as a reference and counter electrode, respectively. EIS test was performed in a frequency range between 100 kHz and 10 mHz at an open-circuit AC amplitude of 5 mV. The cycling stability of electrodes was evaluated at 30 mA cm^−2^ over 10,000 cycles in a three-electrode system. The quasi-solid-state MnO_2_//V_2_O_5_ ASC was assembled out of a CNT/MnO_2_-25/GCC cathode, V_2_O_5_/ECC anode, and filter paper as a separator, and was wrapped by parafilm. The poly(vinyl alcohol)(PVA)/Na_2_SO_4_ gel electrolyte was used and prepared by adding 3 g Na_2_SO_4_ and 3 g PVA in 30 mL of deionized water, followed by stirring at 90 °C for 3 h. The cycling stability of this ASC was evaluated at 15 mA cm^−2^ over 10,000 cycles. The total volume of the ASC is around 0.11 (1 × 1×0.11) cm^3^. The calculation method was described in Supporting Information. Moreover, a pouch-type ASC with a tested area size of 4 (2 × 2) cm^2^ was also assembled.

## Results and Discussion

### Preparation and Characterization of Electrodes

**Fig. 1:**
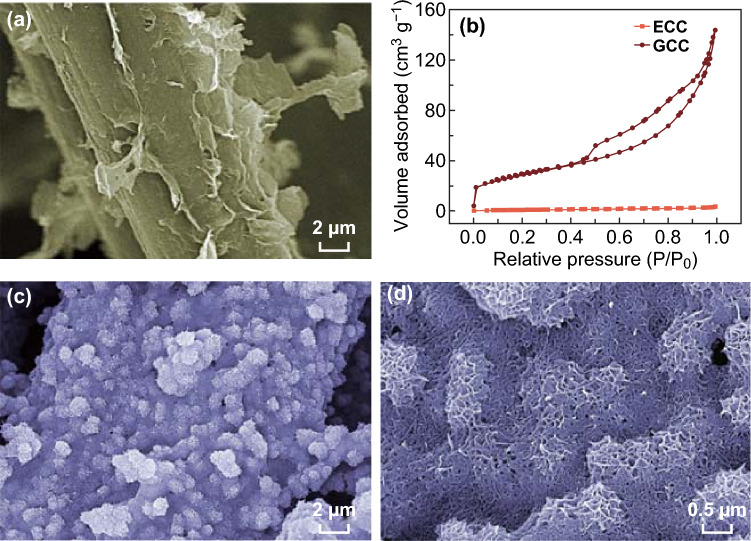
**a** SEM image of GCC. **b** Nitrogen adsorption isotherms of GCC and ECC. **c** Low-resolution, and **d** high-resolution SEM images of CNT/MnO_2_-25/GCC electrodes

As displayed in Scheme 1, 3D porous hierarchical CNT/MnO_2_/GCC electrodes with high MnO_2_ mass loading are prepared by three steps. Firstly, when the positively charged CC modified by PDDA is immersed in the GO solution, negatively charged GO can attach on the CC surface due to the electrostatic attraction [21], and then is reduced to graphene using ascorbic acid as a reducing agent. Secondly, MnO_2_ can be fully covered on GCC via hydrothermal treatment. Thirdly, MnO_2_/GCC is dipped into the CNT solution for 60 s, which allows a uniform coverage of CNT layer on MnO_2_. SEM images of CC (Fig. S1a) and ECC (Fig. S1b) demonstrate bare carbon fiber surfaces, while GCC (Figs. 1a and S1c) displays a rough surface in which graphene nanosheets are tightly anchored on carbon fibers. As shown in Fig. 1b, the specific surface area (SSA) of GCC calculated by a BET method (103.81 m^2^ g^−1^) is larger than that of ECC (4.01 m^2^ g^−1^) due to the presence of graphene on CC. Therefore, GCC can act as an ideal large-surface-area conductive substrate for high MnO_2_ mass loading. After hydrothermal treatment, porous MnO_2_ is uniformly deposited on the substrate (Fig. S1d, e). The intimate interfacial contact between active species and current collector can promote electron transport and reduce interfacial resistance [21, 28]. MnO_2_ has a porous interconnected nanosheet architecture (Fig. S1f) which shortens ion transport pathways and supplies numerous electroactive sites for reversible redox reactions. After dip coating, multiple CNTs are homogeneously covered on MnO_2_ (Fig. 1c, d), beneficial to rapid charge transfer at the electrolyte/electrode interface. Compared to CNT/MnO_2_-25/ECC, densely packed MnO_2_ shows obvious cracks and is loosely deposited on bare ECC (Fig. S2). Moreover, in Fig. S3, the BET surface area of CNT/MnO_2_-25/GCC (60.1 m^2^ g^−1^) is larger than that of CNT/MnO_2_-25/ECC (21.9 m^2^ g^−1^). Hence, the 3D hierarchical CNT/MnO_2_-25/GCC electrode can provide more electrolyte-accessible areas and multiple ion transport channels, which favors the rapid electron and ion transport.

**Fig. 2:**
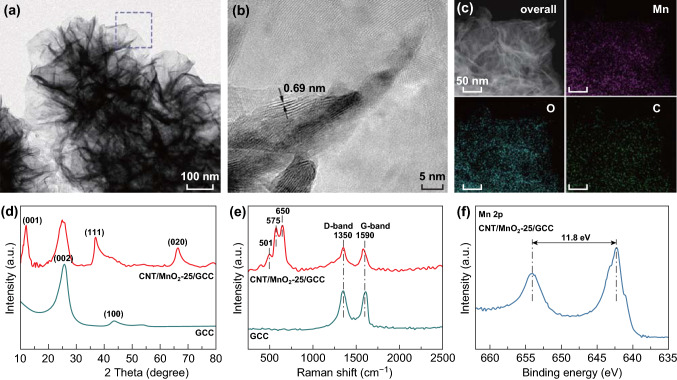
**a** TEM image, **b** HRTEM image, and **c** EDX elemental mapping of the CNT/MnO_2_-25/GCC electrode. **d** XRD spectra, and **e** Raman spectra of GCC and CNT/MnO_2_-25/GCC electrodes. **f** XPS spectrum of Mn 2p peak of the CNT/MnO_2_-25/GCC electrode

TEM images were used to analyze the morphology and crystallinity of MnO_2_. MnO_2_ has a layered nanosheet morphology (Fig. 2a), and it is intertwined with numerous carbon nanotubes (Fig. S4). The lattice fringe with *d*-spacing of 0.69 nm (Fig. 2b) corresponds to the (001) planes of MnO_2_ [2]. The EDX mapping (Fig. 2c) further verifies the existence of Mn, O, and C. XRD pattern of GCC (Fig. 2d) presents two diffraction peaks at 25.7° and 43.4°, corresponding to the (002) and (100) planes of carbon [29]. The characteristic peaks at around 12.2°, 37.1°, and 66.4° in CNT/MnO_2_-25/GCC are indexed to (001), (111), and (020) planes of δ-MnO_2_ (JCPDS No. 42-1317) [4, 30]. Raman spectrum of GCC (Fig. 2e) shows two bands at around 1350 (*D*-band) and 1590 cm^−1^ (*G*-band), while another three bands at around 501, 574, and 650 cm^−1^ in CNT/MnO_2_-25/GCC further demonstrate the characteristics of δ-MnO_2_ [31–33]. XPS survey, Mn 3s, and O 1s spectra of CNT/MnO_2_-25/GCC are displayed in Fig. S5. A spin energy separation of 11.8 eV between Mn 2p_3/2_ (642.3 eV) and Mn 2p_1/2_ (654.1 eV) in Mn 2p spectrum (Fig. 2f) is consistent with previous reports [31–33]. According to 5.4 and 4.7 eV for Mn^3+^ and Mn^4+^, respectively, an energy separation of 4.74 eV in Mn 3s spectrum (Fig. S5b) can estimate the valence state of manganese to be 3.94 [34, 35].

### Electrochemical Performance of Electrodes in a Three-Electrode System

**Fig. 3:**
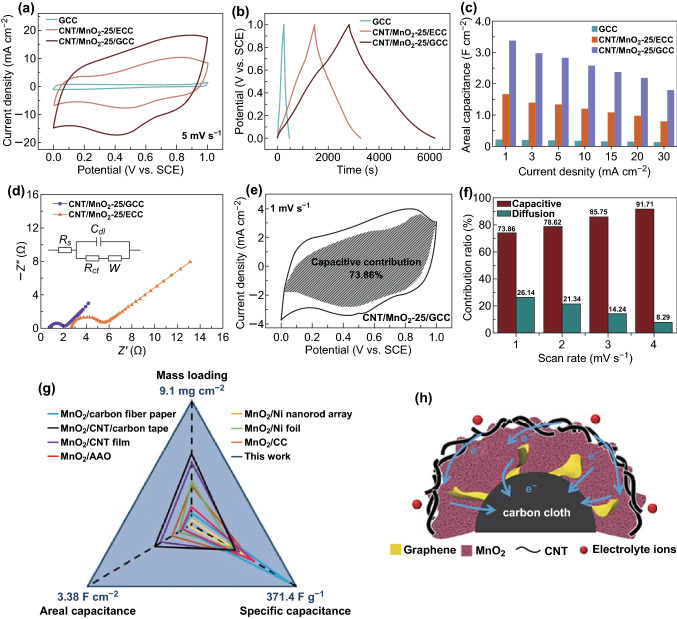
**a** CV curves at a scan rate of 5 mV s^−1^ within a potential window from 0 to 1 V, **b** GCD curves at a current density of 1 mA cm^−2^, and **c** areal capacitances at different current densities from 1 to 30 mA cm^−2^ of GCC, CNT/MnO_2_-25/ECC, and CNT/MnO_2_-25/GCC electrodes. **d** Nyquist plots of CNT/MnO_2_-25/ECC and CNT/MnO_2_-25/GCC electrodes. Inset: the fitted equivalent circuit. *R*_s_, *R*_ct_, *W*, and *C*_dl_ denote equivalent series resistance, charge transfer resistance, Warburg diffusion impedance, and electrochemical double-layer capacitance, respectively. **e** Capacitive contribution (gray region) to the total charge storage at 1 mV s^−1^, and **f** capacitive (red) and diffusion-controlled (green) contribution versus scan rates of the CNT/MnO_2_-25/GCC electrode. **g** Capacitance comparison of the CNT/MnO_2_-25/GCC electrode with previous reports. MnO_2_/carbon fiber paper [22], MnO_2_/CNT/carbon tape [23], MnO_2_/CNT film [24], MnO_2_/anodic aluminum oxide (AAO) [25], MnO_2_/Ni nanorod array [26], MnO_2_/Ni foil [27], MnO_2_/CC [15]. **h** The schematic illustration of the CNT/MnO_2_-25/GCC structure. (Color figure online)

Favorable electrochemical performance of electrodes with high mass loading can be obtained by rationally designing the electrode structure. First of all, the electrochemical property of CNT/MnO_2_-25/GCC and CNT/MnO_2_-25/ECC electrodes is investigated in a three-electrode system to evaluate the crucial role of graphene. A larger integrated area in CV (Fig. 3a) and longer discharging time in GCD curves (Fig. 3b) of the CNT/MnO_2_-25/GCC electrode indicate better electrochemical activity in comparison with GCC and CNT/MnO_2_-25/ECC electrodes. Note that GCC only contributes to a negligible capacity. CV and GCD curves of GCC, CNT/MnO_2_-25/GCC, and CNT/MnO_2_-25/ECC electrodes at various scan rates and current densities are shown in Fig. S6. Rectangular-like CV shapes of the CNT/MnO_2_-25/GCC electrode (Fig. S6a) manifest good capacitive reversibility. Meanwhile, its nearly symmetric GCD profiles and small IR drops in Fig. S6d suggest favorable conductivity and high Coulombic efficiency (over 99.8%). The areal capacitance of CNT/MnO_2_-25/GCC calculated from GCD curves is about 2 and 16.1 times higher than that of CNT/MnO_2_-25/ECC and GCC, i.e., 3.38, 1.66, and 0.21 F cm^−2^ at a current density of 1 mA cm^−2^, respectively. More impressively, in Fig. 3c, the capacitance of the CNT/MnO_2_-25/GCC electrode still remains 1.8 F cm^−2^ at a relatively high current density of 30 mA cm^−2^, which is superior to that of CNT/MnO_2_-25/ECC (0.8 F cm^−2^) and GCC (0.13 F cm^−2^) (Table S1). The presence of graphene on CC increases electrical contact spots between MnO_2_ and current collector, which favors efficient electron transport and leads to outstanding electrochemical performance. Meanwhile, mass loading of MnO_2_ on GCC (9.1 mg) is higher than that of ECC (7.5 mg) because of a larger SSA of GCC. Hence, the specific capacitance of CNT/MnO_2_-25/GCC and CNT/MnO_2_-25/ECC is calculated to be 371.4 and 221.3 F g^−1^, respectively. The better capacitance of CNT/MnO_2_-25/GCC is attributed to high mass loading of MnO_2_, and its intimate combination with the current collector.

Furthermore, cycling stability of the CNT/MnO_2_-25/GCC electrode is also superior to that of CNT/MnO_2_-25/ECC, i.e., 81.4% and 32.5% of the capacitance retention over 10,000 cycles at 30 mA cm^−2^ (Fig. S7), respectively. The decreased capacitance upon cycling is ascribed to the mechanical stress during the repeated charging/discharging process, detachment of electrode materials from the current collector, and dissolution of Mn into the electrolyte [36]. SEM images after cycling disclose that MnO_2_ still closely attaches on the substrate in the CNT/MnO_2_-25/GCC electrode (Fig. S8a), while most MnO_2_ is exfoliated from bare ECC in CNT/MnO_2_-25/ECC (Fig. S8b). These results elucidate that graphene may serve as a buffering network to accommodate the mechanical stress during the cycling process. The EIS test is conducted to estimate charge transfer kinetics. According to the intercept on X-axis and semicircle in Nyquist plots (Fig. 3d), the CNT/MnO_2_-25/GCC electrode reveals a smaller equivalent series resistance (*R*_s_, 0.78 Ω cm^2^) and charge transfer resistance (*R*_ct_, 1.18 Ω cm^2^) compared to those of CNT/MnO_2_-25/ECC (*R*_s_, 2.69 Ω cm^2^; *R*_ct_, 2.5 Ω cm^2^), suggesting better electrical conductivity and rapid charge mobility. This result further convincingly proves that graphene as electrical linkages at the active material/current collector interface can effectively decrease the internal resistance and facilitate the electron transport [37].

Further, the role of CNT on MnO_2_ is evaluated by comparing the electrochemical performance of CNT/MnO_2_-25/GCC and MnO_2_-25/GCC electrodes (Fig. S9). It has been reported that a conductive layer such as CNT or conducting polymers on pseudocapacitive materials can facilitate electron transport [17, 18]. Interestingly, areal capacitances of CNT/MnO_2_-25/GCC (3.38 F cm^−2^) and MnO_2_-25/GCC (3.04 F cm^−2^) electrodes show a small difference at a current density of 1 mA cm^−2^. However, the capacitance retention of the former (65.5%) is 134 times larger than that of the latter (only 0.49%) when the current density is increased from 1 to 20 mA cm^−2^ (Table S2). The inferior rate performance of the MnO_2_-25/GCC electrode is attributed to the low intrinsic conductivity of MnO_2_ and elongated electron transport pathways, while the presence of CNT on top of MnO_2_ offers numerous perpetual and continuous conductive pathways to allow fast electron transport at the electrode/electrolyte interface, and thus effectively enhancing redox reaction kinetics [17, 18]. Meanwhile, compared with CNT/MnO_2_-25/GCC (Table S3), large *R*_s_ (3.97 Ω cm^2^), *R*_ct_ (16.88 Ω cm^2^), and Warburg diffusion impedance (135.3 Ω cm^2^ s^0.5^) derived from the EIS result (Fig. S10) of MnO_2_-25/GCC reveal its inferior redox reaction kinetics and sluggish mass diffusion. Moreover, it has been reported that a conductive protective layer on MnO_2_ may restrict its dissolution into the electrolyte and maintain the structural stability and flexibility [17].

Especially, the charge kinetics of the CNT/MnO_2_-25/GCC electrode can be quantitatively examined by Dunn’s method. At a specific voltage, the current response can be separated to be the capacitive behavior (k_1_*v*) and diffusion-controlled process (k_2_*v*^1/2^) according to Eq. (1) [38]:

1 $$i\left( v \right) = k_{1} v + k_{2} v^{1/2}$$

where k_1_ and k_2_ are the constants. By plotting *v*^1/2^ versus *i/v*^1/2^, k_1_ and k_2_ refer to the slop and y-intercept at a given voltage, respectively [7, 10, 39, 40]. Accordingly, the CV curve of the CNT/MnO_2_-25/GCC electrode in Fig. 3e contrasts the capacitive contribution (gray region) to the total current at a scan rate of 1 mV s^−1^. The percentage of the capacitive contribution is estimated to be 73.86% at a scan rate of 1 mV s^−1^ and is further boosted to be 91.71% at 4 mV s^−1^ (Figs. 3f and S11). It is believed that the hierarchical electrode architecture with favorable electronic/ionic conductivity renders quick charge transfer kinetics and rapid ion transport, leading to a dominating capacitive behavior [7]. Furthermore, the mass loading of MnO_2_ is measured to be 7.4, 9.1, and 14 mg cm^−2^ using 20, 25, and 30 mmol KMnO_4_ during hydrothermal treatment, respectively. The electrochemical performances of CNT/MnO_2_-20/GCC and CNT/MnO_2_-30/GCC electrodes are shown in Figs. S12, S13.

The mass loading of active materials and electrochemical performance of the CNT/MnO_2_-25/GCC electrode are compared with previous reports (Fig. 3g, a comprehensive comparison in Table S4). As illustrated in Fig. 3h, each component in the CNT/MnO_2_-25/GCC electrode plays an important role. Firstly, conductive graphene “bridges” on CC can offer additional surface areas for high MnO_2_ mass loading, guide the uniform distribution of MnO_2_, and enhance its interfacial contact with the substrate, which can reduce the resistance, facilitate electron transport, and maintain structural stability. Secondly, porous nanostructured MnO_2_ could offer short ion diffusion distances and abundant electroactive sites, contributing to exceptional pseudocapacitance. Thirdly, CNT performs as interconnected conductive “highways” to boost the charge transfer, which is beneficial to the rapid ion response at high current densities. Additionally, the free-standing electrode without insulating binders and additives is conducive to obtain high electrochemical performance. Accordingly, the hierarchical structure of the CNT/MnO_2_-25/GCC electrode with high mass loading enables excellent electronic/ionic conductivity, contributing to a large capacitance even at high current densities.

### Electrochemical Performance of the Quasi-Solid-State Flexible ASC

**Fig. 4:**
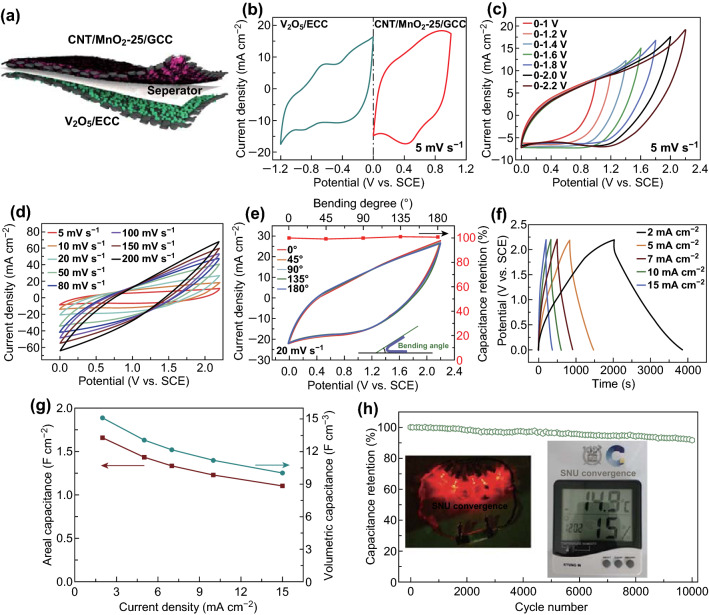
The electrochemical performance of the quasi-solid-state flexible ASC. **a** Schematic illustration of the assembled ASC. **b** CV curves of the cathode and anode at 5 mV s^−1^. **c** CV curves at various voltage windows at 5 mV s^−1^. **d** CV curves at different scan rates from 5 to 80 mV s^−1^. **e** CV curves and capacitance retentions at different bending angles. **f** GCD profiles at different current densities from 2 to 15 mA cm^−2^. **g** Areal and volumetric capacitances at different current densities. **h** Cycling stability at 15 mA cm^−2^ upon 10,000 cycles. Insets: five LEDs and a temperature/humidity indicator powered by the ASC device

A high energy density of flexible supercapacitors is especially important for the practical application. High mass loading of electroactive materials and large potential windows of both anode and cathode contribute to a high energy density. To reveal the practical application, a quasi-solid-state flexible MnO_2_//V_2_O_5_ ASC was assembled with a CNT/MnO_2_-25/GCC cathode, V_2_O_5_/ECC anode, and filter paper separator with the PVA/Na_2_SO_4_ gel electrolyte (Fig. 4a). The characterization and electrochemical performance of prepared V_2_O_5_/ECC are shown in Fig. S14. The method of charge balance between cathode and anode is demonstrated in Supporting Information. Figure 4b displays CV curves of CNT/MnO_2_-25/GCC at a voltage window between 0 and 1 V and V_2_O_5_/ECC between−1.2 and 0 V. Given that, it is anticipated that this ASC could be operated from 0 to 2.2 V. Indeed, Fig. 4c reveals that a high voltage up of the ASC to 2.2 V is achieved, leading to an outstanding energy density. CV profiles of the ASC (Fig. 4d) present a capacitive feature at different scan rates from 5 to 200 mV s^−1^. Particularly, the bending test of the ASC was carried out to evaluate the mechanical stability. As displayed in Fig. 4e, all CV shapes show a small difference, and capacitance retentions remain around 100% at various bending angles (0, 45, 90, 135, and 180 degrees), indicating favorable electrochemical durability and mechanical integrity. More interestingly, Fig. S15a compares the CV curves of MnO_2_//V_2_O_5_ ASC devices with a tested area size of 4 and 1 cm^2^ at a scan rate of 5 mV s^−1^. CV curves of the ASC device (4 cm^2^) at different scan rates are also investigated in Fig. S15b.

**Fig. 5:**
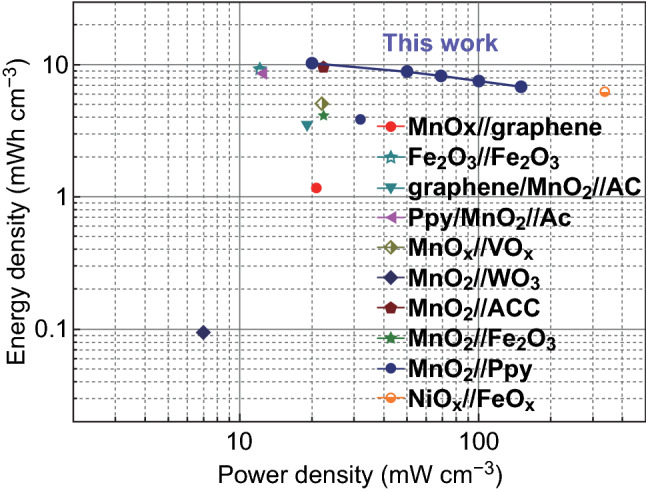
Ragone plots about volumetric energy density and power density of this ASC and other devices with high mass loading of active materials from previous reports. MnO_x_//graphene [14], Fe_2_O_3_//Fe_2_O_3_ [41], graphene//MnO_2_//activated carbon (AC) [42], polypyrrole (Ppy)/MnO_2_//AC [43], MnO_x_//VO_x_ [10], MnO_2_//WO_3_ [44], MnO_2_//activated carbon cloth (ACC) [2], MnO_2_//Fe_2_O_3_ [23], MnO_2_//Ppy [11], and NiO_x_//FeO_x_ [45]

GCD curves (Fig. 4f) of the device demonstrate triangular-like shapes at various current densities, manifesting a reversible capacitive behavior. The areal capacitance based on GCD curves of the device is calculated to be 1.66 F cm^−2^ at a current density of 2 mA cm^−2^, corresponding to a volumetric capacitance of 15.09 F cm^−3^ based on the total volume of the device. The excellent electrochemical property of the MnO_2_//V_2_O_5_ device can be comparable to previous supercapacitors with electrodes at high mass loading (Table S5). Moreover, 66.3% of capacitive retention is obtained when the current density is increased from 2 to 15 mA cm^−2^ (Fig. 4g). According to the Nyquist plot (Fig. S16), the MnO_2_//V_2_O_5_ ASC demonstrates a small equivalent series resistance (*R*_s_, 1.26 Ω cm^2^) and charge transfer resistance (*R*_ct_, 7.9 Ω cm^2^). Further, the MnO_2_//V_2_O_5_ ASC demonstrates excellent structural stability in which 91.8% of the original capacitance remains after 10,000 cycles at 15 mA cm^−2^ (Fig. 4h). Five parallel light-emitting diodes (2.0 V) and a temperature/humidity indicator (1.5 V) can be powered by one MnO_2_//V_2_O_5_ ASC device (insets in Fig. 4h), suggesting its potential for practical application. More importantly, as illustrated in Ragone plots (Fig. 5), the MnO_2_//V_2_O_5_ ASC delivers a high volumetric energy density of 10.18 mWh cm^−3^ at 20 mW cm^−3^, which are compared with other supercapacitors with high mass loading in previous reports. According to the abovementioned equation of *E* = 0.5 *CV*^2^, it is believed that the high energy density of the MnO_2_//V_2_O_5_ ASC is originated from the high capacitance of electrodes together with a wide cell voltage of 2.2 V.

The difference in this study from previous Mn-based papers is explained as follows. Firstly, the small mass loading of active materials (< 1 mg cm^−2^) is generally adopted in previous reports, which is not favorable for the commercial application. In this study, high mass loading of active materials is applied to achieve outstanding electrochemical performance. Secondly, to address problems caused by high MnO_2_ mass loading, various reported strategies such as electrochemical method [7], metal atom doping [8, 9], and Ostwald ripening [10] were used to alter the crystalline property, which can increase the electrochemical activity of electrodes. Herein, we would like to achieve high electrochemical performance at high MnO_2_ mass loading via the rational structure design of the electrode. Furthermore, because of the high capacitance of cathode and anode along with a wide voltage window (2.2 V), the assembled MnO_2_//V_2_O_5_ supercapacitor obtains a high energy density of 10.18 mWh cm^−3^, which is higher than some supercapacitors in previous reports.

## Conclusion

In summary, a 3D hierarchical conductive CNT/high mass loading MnO_2_/GCC electrode is prepared for supercapacitor. The employment of conductive graphene “bridges” on CC and CNT “highways” on MnO_2_ in the CNT/MnO_2_/GCC electrode can effectively enhance the mass loading of MnO_2_, reduce the internal resistance, improve the structural stability, and facilitate the charge transfer, greatly optimizing the capacitive utilization of MnO_2_. Due to the synergistic effect of three components, the well-designed CNT/MnO_2_/GCC electrode provides sufficient electroactive spots, multiple ion transport pathways, superior charge collection ability, and rapid redox reaction kinetics. Hence, the favorable electronic/ionic conductivity of the CNT/MnO_2_/GCC electrode contributes to a remarkable areal capacitance without compromising rate capability even at high mass loading. An outstanding energy density of the flexible MnO_2_//V_2_O_5_ asymmetric supercapacitors indicates its practical application, which results from high mass loading of electrodes and a wide voltage window.

## Electronic Supplementary Material

Below is the link to the electronic supplementary material.

Supplementary material 1 (PDF 1838 kb)
