# Supplementary material for: CNT/High Mass Loading MnO2/Graphene-Grafted Carbon Cloth Electrodes for High-Energy Asymmetric Supercapacitors
Source: Nanomicro Lett. 2019 Oct 17;11:88. doi: 10.1007/s40820-019-0316-7 (PMC7770775; doi:10.1007/s40820-019-0316-7)
Supplement: Supplementary file 1 — Supplementary material 1 (PDF 1838 kb) [file 40820_2019_316_MOESM1_ESM.pdf]

Supporting Information for

CNT/High Mass Loading MnO_2/Graphene-grafted Carbon Cloth Electrodes for High-energy Asymmetric Supercapacitors

Lulu Lyu[1], Kwang-dong Seong[1], Jong Min Kim[1], Wang Zhang[2], Xuanzhen Jin[1], Dae Kyom Kim[1], Youngmoo Jeon[1], Jeongmin Kang[1],[a] Yuanzhe Piao[1, 3, *]

[1]Graduate School of Convergence Science and Technology, Seoul National University, Seoul, 151-742, Republic of Korea

[2]School of Chemistry and Chemical Engineering, Yangzhou University, Yangzhou, 225002 Jiangsu, People's Republic of China

[3]Advanced Institutes of Convergence Technology, Suwon, 443-270, Republic of Korea

*Corresponding author. E-mail: parkat9@snu.ac.kr (Y. Piao)

S1 Preparation of Graphene Oxide

Graphene oxide (GO) was synthesized by a modified Hummers' method. In a typical synthesis, 1.5 g of graphite, 1.25 g of $K_2S_2O_8$ and 2.5 g of P_2O_5 were dissolved in 40 mL of H_2SO_4 solution, and the mixture was stirred at 95 °C for 5 h. Then, the powder was washed with copious water and dried in an oven. Afterward, the dried powder was dispersed in 60 mL of H_2SO_4 solution and followed by adding 7.5 g of $KMnO_4$ slowly. After stirring at 85 °C for 4 h, 8 mL of H_2O_2 was poured to the above mixture. Then, the resultant was washed with HCl aqueous solution and water thoroughly and followed by freeze-drying. A 2 mg mL^{-1} of GO solution was prepared by dispersing 10 mg of GO in 5 mL of deionized water and sonicated for 2 h.

S2 Preparation of CNT Solution

The commercial multi-walled carbon nanotubes were refluxed with a mixture of H_2SO_4 and HNO_3 solution (3:1, v/v) at 70 °C for 3 h. The resultant was washed thoroughly with copious water and dried in an oven. Next, 0.5 wt% CNT solution was prepared by diluting CNT powder in deionized water.

S3 Method of Calculation

S3.1 The Areal and Specific Capacitance for a Single Electrode in a Three-electrode Test

The areal and specific capacitance of a single electrode in a three-electrode test can be calculated from galvanostatic charge-discharge curves based on Eqs. S1 and S2:

$$C_a = \frac{I \times t}{\Delta V \times A} \quad (S1)$$

$$C_m = \frac{1000 C_a}{m} \quad (S2)$$

in which C_a (F cm^{-2}) and C_m (F g^{-1}) refer to the areal capacitance and specific capacitance of a single electrode, respectively. I is the current (A); t is the discharging time (s); ΔV is the voltage range (V); A is the working area of the electrode (cm^2); m is the mass loading of MnO_2 (mg) in an electrode.

S3.2 Areal and Volumetric Capacitance of the Quasi-solid-state Flexible MnO_2//V_2O_5 Asymmetric Supercapacitor

$$C_a = \frac{I \times t}{\Delta V \times A} \qquad \text{(S3)}$$

$$C_v = \frac{C_a}{V} \qquad \text{(S4)}$$

in which C_a (F cm^{-2}) and C_v (F cm^{-3}) refer to the areal capacitance and volumetric capacitance of the MnO_2//V_2O_5 asymmetric supercapacitor, respectively. I is the current (A); t is the discharging time (s); ΔV is the voltage range of the asymmetric supercapacitor (V); A is the tested area of the asymmetric supercapacitor (cm^2); V is the total volume of the asymmetric supercapacitor (cm^3).

S3.3 Energy and Power Density of the Quasi-solid-state Flexible MnO_2//V_2O_5 Asymmetric Supercapacitor

The volumetric energy density and power density of the assembled MnO_2//V_2O_5 supercapacitor can be evaluated from the following Eqs. S5 and S6:

$$E_v = \frac{1000 \times C_v \mathrm{V}^2}{2 \times 3600} \qquad \text{(S5)}$$

$$P_v = \frac{3600 \times E_v}{t} \qquad \text{(S6)}$$

in which E_v (mWh cm^{-3}) and P_v (mW cm^{-3}) refer to the volumetric energy density and power density, respectively; C_v is the volumetric capacitance (F cm^{-3}); t is the discharging time (s); V is the voltage range (V).

S3.4 Charge Balance for the Quasi-solid-state MnO_2//V_2O_5 Flexible ASC

The stored charge (Q) in the cathode and anode can be calculated based on Eqs. S7 and S8:

$$Q_+ = C_{a,+} \times \Delta V \times A_+ \qquad \text{(S7)}$$

$$Q_- = C_{a,-} \times \Delta V \times A_- \qquad \text{(S8)}$$

In which C, ΔV, and A are the areal capacitance, voltage window, and working area of each electrode, respectively.

When Q_+=Q_-, the optimum electrochemical performance of the ASC can be achieved. Therefore, the areal capacitance of the anode and cathode should correspond to Eq. S9:

$$\frac{C_{a,-}}{C_{a,+}} = \frac{\Delta V_+ \times A_+}{\Delta V_- \times A_-} \qquad \text{(S9)}$$

The ΔV of V_2O_5/ECC (anode) and CNT/MnO_2-25/GCC (cathode) is 1.2 and 1.0 V, respectively. The working area of each electrode is 1.0 cm^2. Therefore, according to Eq. S9, the capacitance ratio of anode and cathode is about 0.83. Because the areal capacitance of the CNT/MnO_2-25/GCC electrode is 3.38 F cm^{-2} at a current density of 1 mA cm^{-2}, the areal capacitance of V_2O_5/ECC is expected to be 2.8 F cm^{-2} at 1 mA cm^{-2}. The mass loading of active materials of cathode and anode is 9.1 and 11.8 mg cm^{-2}.

S4 Supplementary Figures and Tables

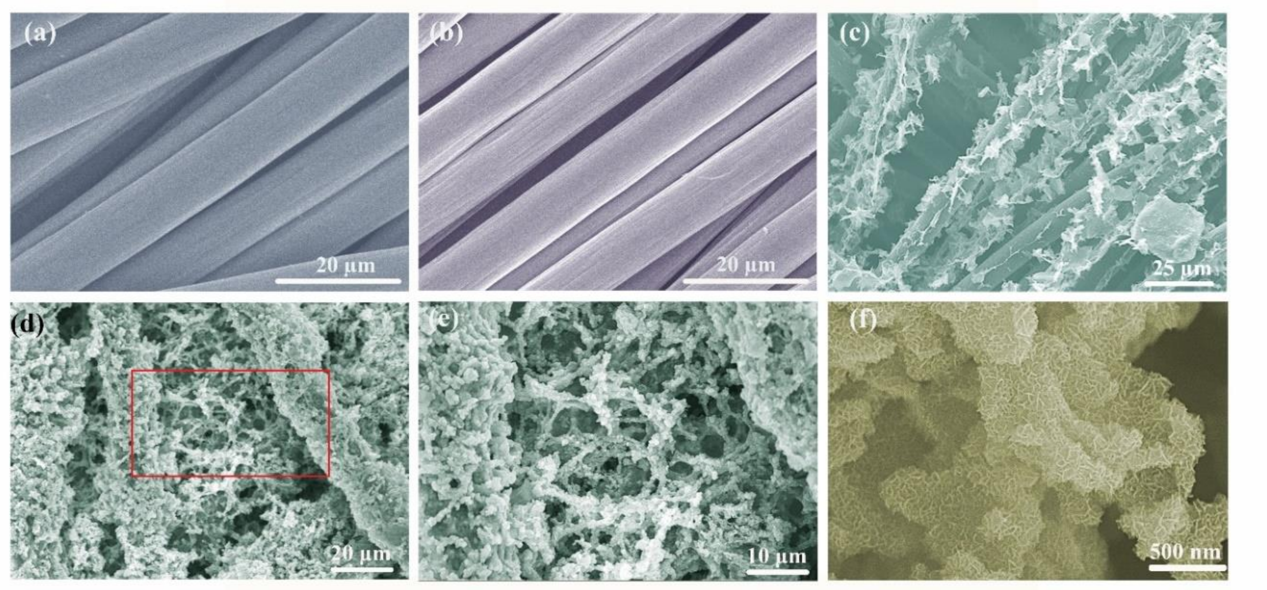

Fig. S1 SEM images of **a** pristine CC, **b** ECC, **c** GCC, and **d, e** MnO_2/GCC electrodes. **f** High-resolution SEM image of the MnO_2/GCC electrode

Note to Fig. S1 Pristine CC (Fig. S1a) indicates aligned carbon fibers with a smooth surface, while ECC (Fig. S1b) shows scratches on carbon fibers due to the electrochemical activation. Fig. S1e shows an enlarged SEM image in the red square of Fig. S1d, which reveals that MnO_2 is not only fully covered on carbon fibers, and some MnO_2 exists gaps and voids between fibers. MnO_2 reveals an interconnected nanosheet morphology in Fig. S1f.

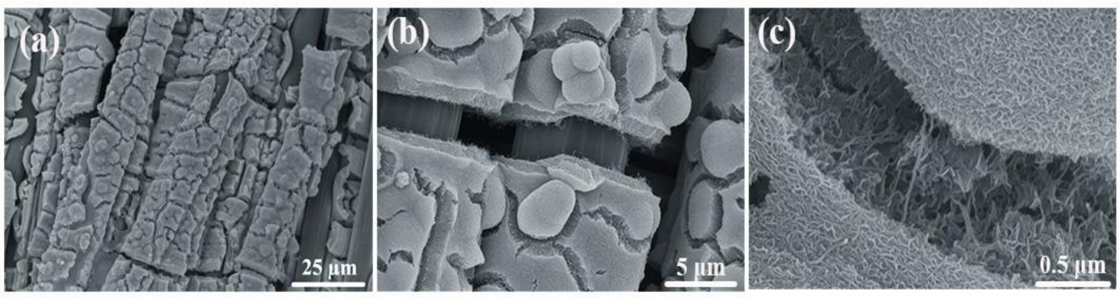

Fig. S2 SEM images of the CNT/MnO_2/ECC electrode

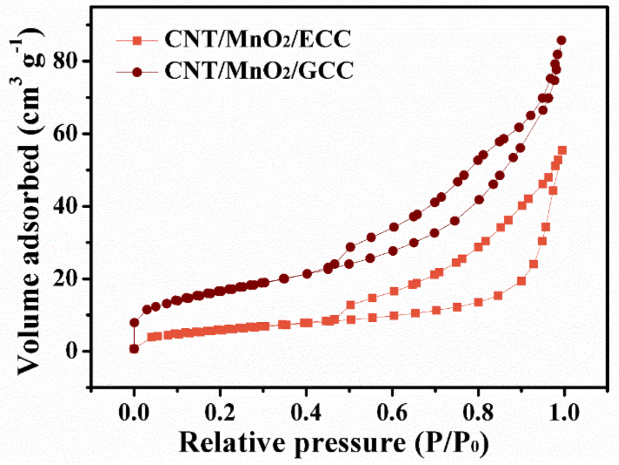

Fig. S3 Nitrogen adsorption isotherms of CNT/MnO_2-25/ECC and CNT/MnO_2-25/GCC electrodes

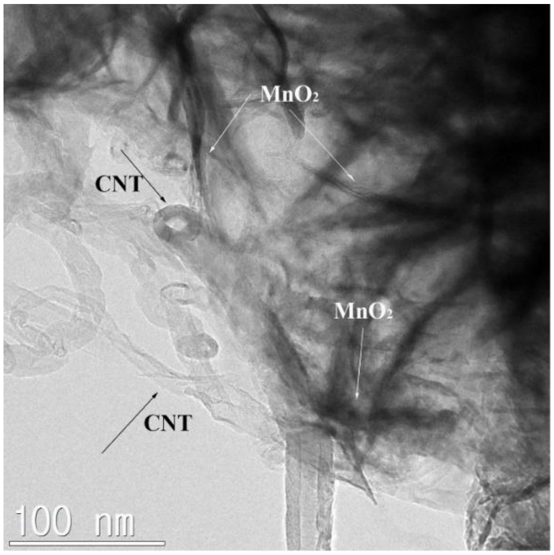

Fig. S4 The TEM image of the CNT/MnO_2-25/GCC sample

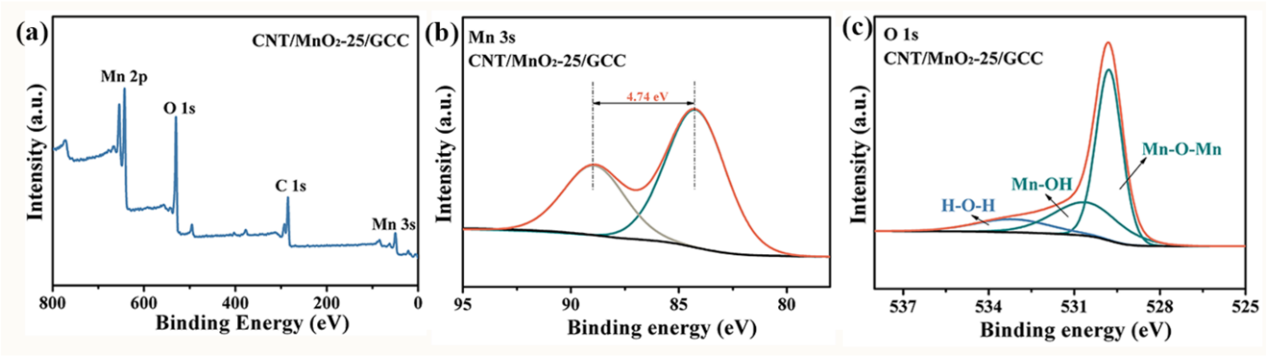

Fig. S5 **a** XPS survey spectrum, **b** Mn 3s, and **c** O 1s of the CNT/MnO_2-25/GCC electrode

<u>Note to Fig. S5</u> XPS survey spectrum of the CNT/MnO_2/GCC electrode in Fig. S5a reveals the existence of C, Mn, and O. The O 1s spectrum in Fig. S5c can be fitted with three peaks at 529.79, 530.59, and 533.16 eV, referring to Mn-O-Mn, Mn-OH, and H-O-H, respectively [S1]. The structural water is beneficial to rapid ion diffusion within oxide lattice [S2].

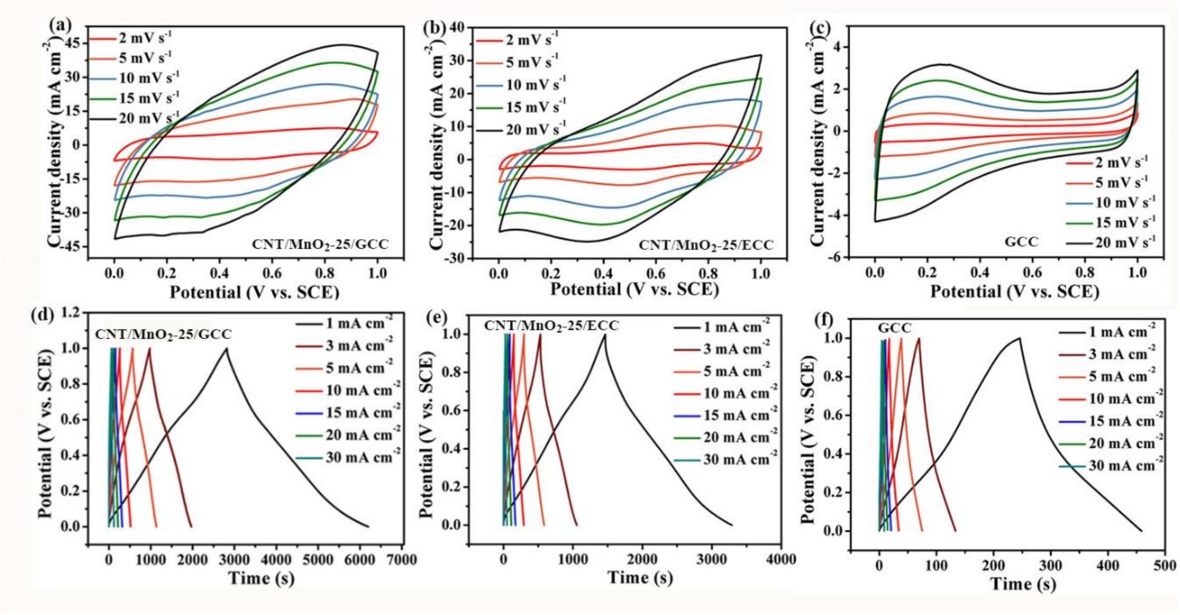

Fig. S6 CV curves of **a** CNT/MnO_2-25/GCC, **b** CNT/MnO_2-25/ECC, and **c** GCC electrodes at different scan rates from 2 to 20 mV s^{-1}. GCD curves of **d** CNT/MnO_2-25/GCC, **e** CNT/MnO_2-25/ECC, and **f** GCC electrodes at different current densities from 1 to 30 mA cm^{-2}

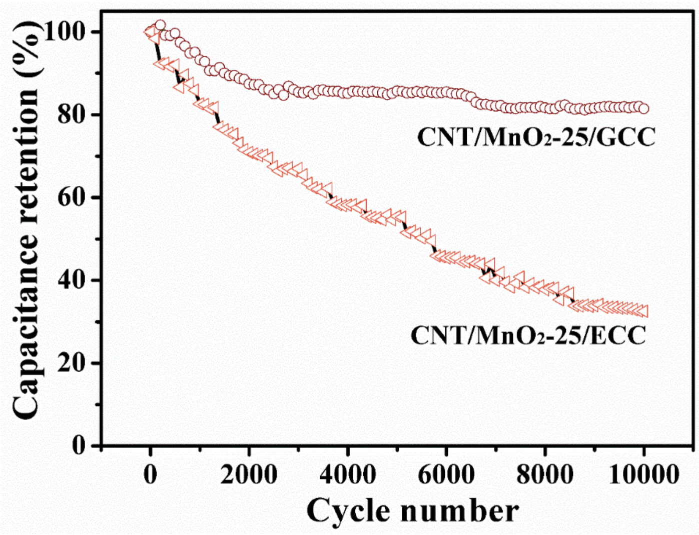

Fig. S7 Cycling stability of CNT/MnO_2-25/ECC and CNT/MnO_2-25/GCC for 10000 cycles electrodes at a current density of 30 mA cm^{-1} in a three-electrode system

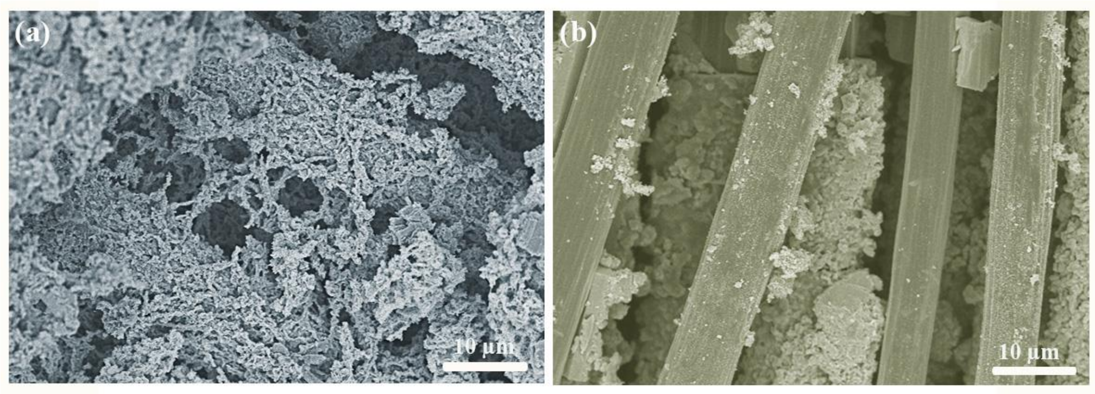

Fig. S8 SEM images of **a** CNT/MnO_2-25/GCC, and **b** CNT/MnO_2-25/ECC electrodes after 10000 cycles

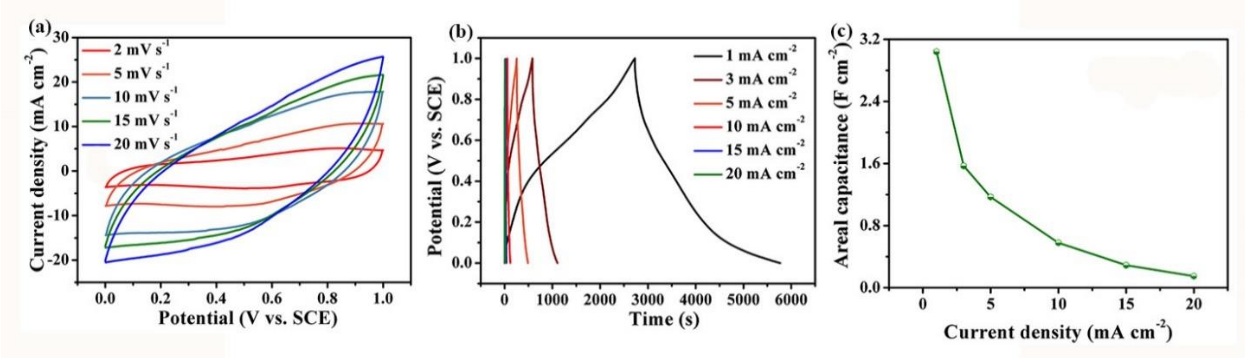

Fig. S9 **a** CV curves at different scan rates from 2 to 20 mV s^{-1}, **b** GCD curves, and **c** capacitances at different current densities from 1 to 20 mA cm^{-2} of the MnO_2-25/GCC electrode

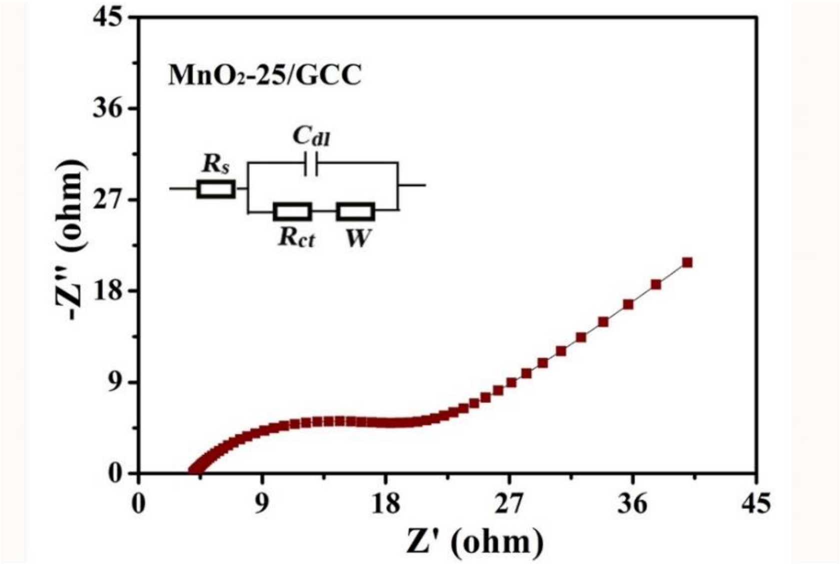

Fig. S10 Nyquist plot of the MnO_2-25/GCC electrode

Note to Fig. S10 The inset displays the fitted equivalent circuit. In the high frequency region, the equivalent series resistance (R_s) is composed of the intrinsic resistance, electrolyte ionic resistance and interfacial resistance between active materials and the current collector. The value of R_s can be obtained from the X-intercept of the EIS curve. In the middle frequency region, the diameter of the semicircle indicates a charge transfer resistance (R_{ct}). In the low frequency region, the Warburg impedance (W) is related to ion diffusion at the electrode/electrolyte interface.

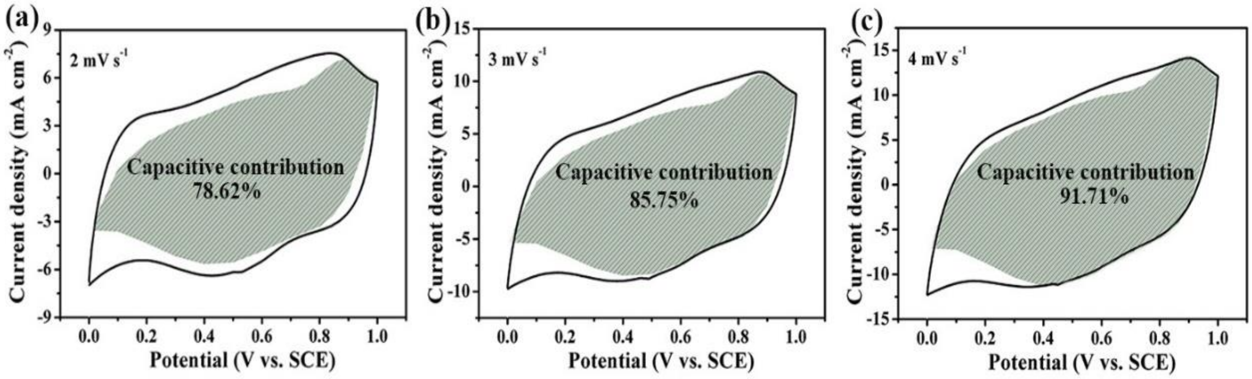

Fig. S11 The capacitive contribution to the total charge storage of the CNT/MnO_2-25/GCC electrode at a scan rate of **a** 2 mV s^{-1}, **b** 3 mV s^{-1}, and **c** 4 mV s^{-1}

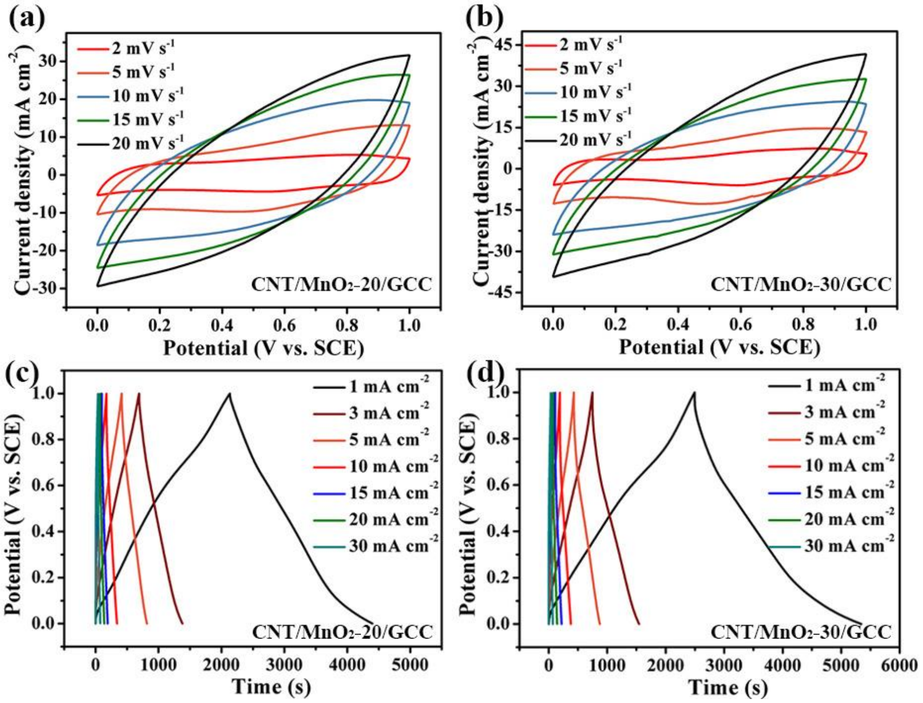

Fig. S12 CV curves of **a** CNT/MnO_2-20/GCC, and **b** CNT/MnO_2-30/GCC electrodes at different scan rates from 2 to 20 mV s^{-1}. GCD curves of **c** CNT/MnO_2-20/GCC, and **d** CNT/MnO_2-30/GCC electrodes at different current densities from 1 to 30 mA cm^{-2}

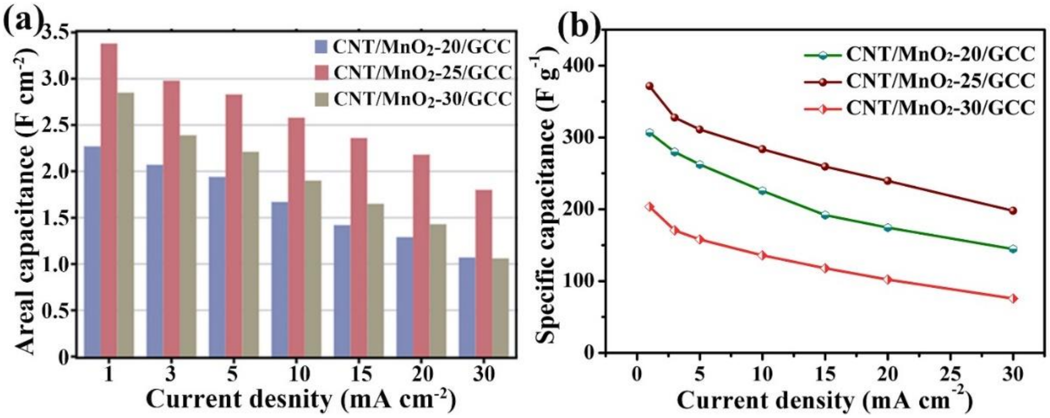

Fig. S13 a Areal capacitances, and **b** specific capacitances calculated from GCD profiles of CNT/MnO_2-20/GCC, CNT/MnO_2-25/GCC and CNT/MnO_2-30/GCC at different current densities from 1 to 30 mA cm^{-2}

<u>**Note to Fig. S13**</u> The areal capacitances of CNT/MnO_2-20/GCC, CNT/MnO_2-25/GCC and CNT/MnO_2-30/GCC electrodes are calculated to be 2.27, 3.38, and 2.85 mF cm^{-2} at 1 mA cm^{-2}, corresponding to a specific capacitance of 306.8, 371.4, and 203.6 F g^{-1}. About 47.1%, 53.3%, and 37.2% of the initial capacitance are preserved from 1 to 30 mA cm^{-1}, respectively. The decreased performance of the CNT/MnO_2-30/GCC electrode indicates that ultrahigh MnO_2 loading may reduce electroactive areas and increase the electrode resistance.

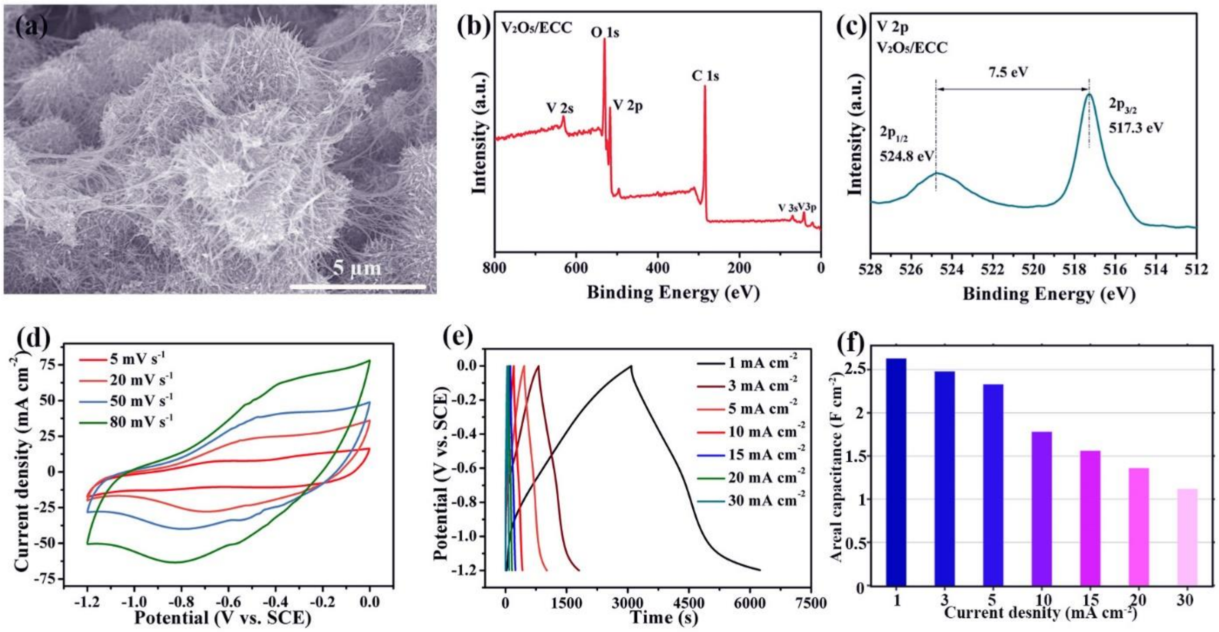

Fig. S14 The characterization and electrochemical performance of the V_2O_5/ECC electrode. **a** SEM image. **b** XPS survey spectra. **c** V 2p spectrum. **d** CV curves at different scan rates from 5 to 80 mV s^{-1}. **e** GCD profiles at different current densities from 1 to 30 mA cm^{-2}. **f** The areal capacitance calculated from GCD curves at different current densities

Note to Fig. S14 Vanadium oxide was electrochemically deposited on ECC. The SEM image of vanadium oxide in Fig. S14a indicates an interconnected urchin-like morphology. XPS spectrum of the V_2O_5/ECC electrode in Fig. S14b reveals the co-existence of V, O, and C. Moreover, in Fig. S14c, a spin energy separation (7.5 eV) between V $2p_{1/2}$ (524.8 eV) and V $2p_{3/2}$ (517.3 eV) discloses the valence state of vanadium to be 5. Meanwhile, the electrochemical property of the V_2O_5/ECC electrode was evaluated by CV and GCD under a three-electrode configuration with SCE and Pt as the reference and counter electrode in 1 M Na_2SO_4 electrolyte. Figure S14d demonstrates CV curves of the V_2O_5/ECC electrode at different scan rates of 5 to 80 mV s^{-1} within a voltage window from -1.2 to 0 V. Figure S14e displays GCD profiles at different densities from 1 to 30 mA cm^{-2}. The V_2O_5/ECC electrode can deliver a high areal capacitance of 2.63 and 1.12 F cm^{-2} at a current density of 1 and 30 mA cm^{-2}, respectively (Fig. S14f). 42.6% of the capacitance remains. The excellent capacitance and rate capability of the V_2O_5/ECC electrode are attributed to the high mass loading of active materials (11.8 mg cm^{-2}) and hierarchical construction.

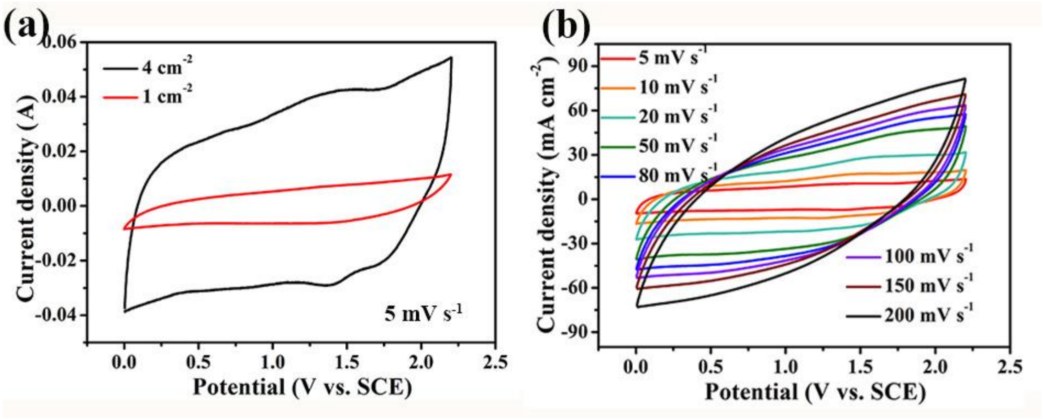

Fig. S15 a The comparison of CV curves of $MnO_2//V_2O_5$ ASC devices with a tested area of 4 and 1 cm^2 at a scan rate of 5 mV s^{-1}. **b** CV curves of the $MnO_2//V_2O_5$ ASC device with a tested area of 4 cm^2 at different scan rates

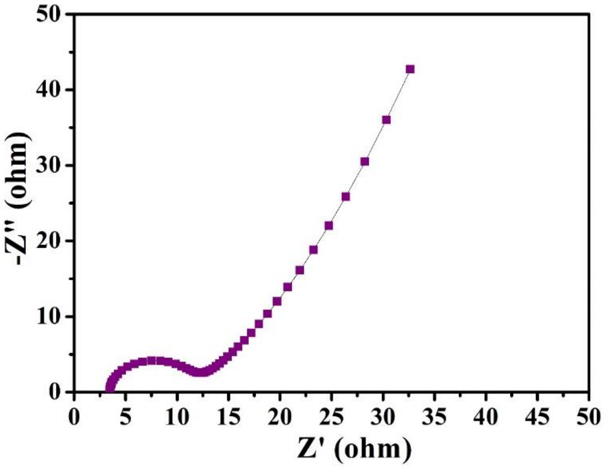

Fig. S16 Nyquist plot of the $MnO_2//V_2O_5$ ASC

Table S1 Areal capacitances of GCC, CNT/MnO_2-25/GCC and CNT/MnO_2-25/ECC electrodes at different current densities

Samples	Current density (mA cm^{-2})								Capacitance retention (1~30 mA cm^{-2})
	1	3	5	7	10	15	20	30	
	Areal capacitance (F cm^{-2})								
GCC	0.21	0.19	0.18	0.18	0.17	0.15	0.14	0.13	62%
CNT/MnO_2-25/GCC	3.38	2.98	2.83	2.73	2.58	2.36	2.18	1.8	53.3%
CNT/MnO_2-25/ECC	1.66	1.4	1.33	1.27	1.2	1.08	0.98	0.8	48%

Table S2 Areal capacitances of CNT/MnO_2-25/GCC and MnO_2-25/GCC electrodes at different current densities

Samples	Current density (mA cm^{-2})								Capacitance retention
	1	3	5	7	10	15	20	30	
	Areal capacitance (F cm^{-2})								
CNT/MnO_2-25/GCC	3.38	2.98	2.83	2.73	2.58	2.36	2.18	1.8	76.3% (1~10 mA cm^{-2})<br>65.5% (1~20 mA cm^{-2})<br>53.3% (1~30 mA cm^{-2})
MnO_2-25/GCC	3.04	1.57	1.17	0.9	0.58	0.29	0.15	/	0.49% (1~20 mA cm^{-2})

Table S3 R_s, R_{ct}, and W derived from the equivalent circuit fitted with the Nyquist plots of CNT/MnO_2-25/GCC and MnO_2-25/GCC electrodes

Samples	R_s (Ω cm^2)	R_{ct} (Ω cm^2)	Warburg diffusion impedance (Ω cm^2 s$^{0.5}$)
CNT/MnO_2-25/GCC	0.78	1.18	0.4
MnO_2-25/GCC	3.97	16.88	135.3

Table S4 Comparison of the electrochemical performance of the CNT/MnO_2-25/GCC electrode with other highly loaded MnO_2 in previous reports

Samples	Mass loading (mg)	Areal capacitance	Rate performance	Refs.
MnO_2	3	651.7 mF cm^{-2} at 1 mA cm^{-2}	56.8% (1-15 mA cm^{-2}, 15 times)	[S3]
MnO_2	3.4	557 mF cm^{-2} at 5 mA cm^{-2}	39.9% (5-100 mA cm^{-2}, 20 times)	[S4]
graphene/MnO_2	3.7	897 mF cm^{-2} at 0.05 A g^{-1}	50.1% (0.05-0.5 A g^{-1}, 10 times)	[S5]
MnO_xN_y/rGO[1]/cellulose	6.02	2743.7 mF cm^{-2} at 1.1 A g^{-1}	70% (1.1-16.2 A g^{-1}, 15 times)	[S6]
MnO_2	8.1	202 mF cm^{-2} at 0.1 mA cm^{-2}	33.7% (0.1-2 mA cm^{-2}, 20 times)	[S7]
MnO_2	5	964 mF cm^{-2} at 1 mA cm^{-2}	81% (1-10 mA cm^{-2}, 10 times)	[S8]
MnO_2	2.441	540 mF cm^{-2} at 0.1 mA cm^{-2}	68.1% (0.1-1.5 mA cm^{-2}, 15 times)	[S9]
PPy[2]/MnO_2	5.4	2.45 F cm^{-2} at 0.2 mA cm^{-2}	34% (0.2-10 mA cm^{-2}, 50 times)	[S10]
MnO_2	6.6	1.1 F cm^{-2} at 1 mA cm^{-2}	63.6% (1-15 mA cm^{-2}, 15 times)	[S11]
MnO_2	18	2790 mF cm^{-2} at 2 mA cm^{-2}	31% (2-20 mA cm^{-2}, 10 times)	[S12]
MnO_2/CNF[3]	3.1	525 mF cm^{-2} at 3 mA cm^{-2}	54% (3-30 mA cm^{-2}, 10 times)	[S13]

MnO_2/CNT	6.4	1.0 F cm^{-2} at 1.28 mA cm^{-2}	77% (1.28-128 mA cm^{-2}, 100 times)	[S14]
MnO_2	4.9	235 mF cm^{-2} at 1 mA cm^{-2}	45.2% (1-5 mA cm^{-2}, 5 times)	[S15]
MnO_2	16.99	2500 mF cm^{-2} at 1 mV s^{-1}	/	[S16]
MnO_2	13.6	3.18 F cm^{-2} at 1 mV s^{-1}	31.4% (1-40 mA cm^{-2}, 40 times)	[S17]
Fe-doped MnO_2	5	1335 mF at 0.1 A g^{-1}	68.6 % (0.1-5 A g^{-1}, 50 times)	[S18]
MnO_2	4.5	2088 mF cm^{-2} at 4 mA cm^{-2}	46.3% (4-15 mA cm^{-2}, 3.75 times)	[S19]
**MnO_2**	**9.1**	**3.38 F cm^{-2} at 1 mA cm^{-2}**	**76.3% (1-10 mA cm^{-2}, 10 times)**<br>**64.5% (1-20 mA cm^{-2}, 20 times)**<br>**53.1% (1-30 mA cm^{-2}, 30 times)**	**This work**

[1]reduced graphene oxide; [2]polypyrrole; [3]carbon nanofiber

Table S5 Comparison of the volumetric energy density the MnO_2//V_2O_5 ASC with other supercapacitor devices

Electrode materials	Electrolyte	Voltage window (V)	Capacitance	Volumetric energy density (mWh cm^{-3})	Refs.
MnO_2//V_2O_5	PVA/Na_2SO_4	0-2	14.41 F cm^{-3} at 1 mA cm^{-2}	8.25	[S20]
MnO_x//VO_x	LiCl	0-2.2	7.46 F cm^{-3} at 2 mA cm^{-2}	5	[S2]
Fe_2O_3//Fe_2O_3	3M $LiNO_3$	0-1.8	1531 mF cm^{-2} at 1 A cm^{-2}	9.2	[S21]
MnO_2//MnO_2	1M Na_2SO_4	0-1.6	160 mF cm^{-2} at 1 A cm^{-2}	5.71	[S22]
Graphene/MnO_2//AC	PVA/LiCl	0-1.8	8.15 F cm^{-3} at 1 mA cm^{-2}	3.5	[S23]

$MnO_2//Fe_2O_3$	PVA/$LiClO_4$	0-2.1	6.8 F cm^{-3} at 0.5 mA cm^{-2}	4.1	[S11]
MnO_x//graphene	PVA/Na_2SO_4	0-1.8	2.57 F cm^{-3} at 1 mA cm^{-2}	1.16	[S24]
Fe-doped MnO_2//Fe-doped MnO_2	PVA/KOH	0-0.8	14.65 F cm^{-3} at 1 mA cm^{-2}	1.3	[S18]
MnO_2//h-WO_3	1 M Na_2SO_4	0-1.4	350 mF cm^{-3} at 1 mA cm^{-2}	0.096	[S15]
**$MnO_2//V_2O_5$**	**PVA/Na_2SO_4**	**0-2.2**	**15.09 F cm^{-3} at 2 mA cm^{-2}**	**10.18**	**This work**

Supplementary References

[S1]D.P. Dubal, D.S. Dhawale, R.R. Salunkhe, C.D. Lokhande, Conversion of chemically prepared interlocked cubelike Mn_3O_4 to birnessite MnO_2 using electrochemical cycling. J. Electrochem. Soc. **157**, A812–A817 (2010). https://doi.org/10.1149/1.3428675

[S2]Y. Song, T. Liu, B. Yao, M. Li, T. Kou et al., Ostwald ripening improves rate capability of high mass loading manganese oxide for supercapacitors. ACS Energy Lett. **2**, 1752–1759 (2017). https://doi.org/10.1021/acsenergylett.7b00405

[S3]X. Liu, C. Guan, Y. Hu, L. Zhang, A.M. Elshahawy, J. Wang, 2D metal–organic frameworks derived nanocarbon arrays for substrate enhancement in flexible supercapacitors. Small **14**, 1702641 (2018). https://doi.org/10.1002/smll.201702641

[S4]S.H. Kazemi, M.A. Kiani, M. Ghaemmaghami, H. Kazemi, Nano-architectured MnO_2 electrodeposited on the Cu-decorated Nickel foam substrate as supercapacitor electrode with excellent areal capacitance. Electrochim. Acta **197**, 107–116 (2016). https://doi.org/10.1016/j.electacta.2016.03.063

[S5]A. Sumboja, C.Y. Foo, X. Wang, P.S. Lee, Large areal mass, flexible and free-standing reduced graphene oxide/manganese dioxide paper for asymmetric supercapacitor device. Adv. Mater. **25**, 2809–2815(2013). https://doi.org/10.1002/adma.201205064

[S6]Q. Zheng, R. Xie, L. Fang, Z. Cai, Z. Ma, S. Gong, Oxygen-deficient and nitrogen-doped MnO_2 nanowire-reduced graphene oxide-cellulose nanofibril aerogel electrodes for high-performance asymmetric supercapacitors. J. Mater. Chem. A **6**, 24407–24417 (2018). https://doi.org/10.1039/C8TA09374A

[S7]C. Wan, Y. Jiao, D. Liang, Y. Wu, J. Li, A high-performance, all-textile and spirally wound

asymmetric supercapacitors based on core–sheath structured MnO_2 nanoribbons and cotton-derived carbon cloth, Electrochim. Acta **285**, 262–271 (2018). https://doi.org/10.1016/j.electacta.2018.07.036

[S8] L. Wang, M. Huang, S. Chen, L. Kang, X. He et al., δ-MnO_2 nanofiber/single-walled carbon nanotube hybrid film for all-solid-state flexible supercapacitors with high performance. J. Mater. Chem. A **5**, 19107–19115 (2017). https://doi.org/10.1039/C7TA04712F

[S9] Y. Gao, Y. Lin, Z. Peng, Q. Zhou, Z. Fan, Accelerating ion diffusion with unique three-dimensionally interconnected nanopores for self-membrane high-performance pseudocapacitors. Nanoscale **9**, 18311–18317 (2017). https://doi.org/10.1039/C7NR06234F

[S10] J. Tao, N. Liu, L. Li, J. Su, Y. Gao, Hierarchical nanostructures of polypyrrole@MnO_2 composite electrodes for high performance solid-state asymmetric supercapacitors. Nanoscale **6**, 2922–2928 (2014). https://doi.org/10.1039/c3nr05845j

[S11] Y. Zhang, X. Yuan, W. Lu, Y. Yan, J. Zhu, T.W. Chou, MnO_2 based sandwich structure electrode for supercapacitor with large voltage window and high mass loading. Chem. Eng. J. **368**, 525–532 (2019). https://doi.org/10.1016/j.cej.2019.02.206

[S12] J. Yang, L. Lian, H. Ruan, F. Xie, M. Wei, Nanostructured porous MnO_2 on Ni foam substrate with a high mass loading via a CV electrodeposition route for supercapacitor application. Electrochim. Acta **136**, 189–194 (2014). https://doi.org/10.1016/j.electacta.2014.05.074

[S13] H. Xu, X. Hu, H. Yang, Y. Sun, C. Hu, Y. Huang, Flexible asymmetric micro-supercapacitors based on Bi_2O_3 and MnO_2 nanoflowers: Larger areal mass promises higher energy density. Adv. Energy Mater. **5**, 1401882 (2015). https://doi.org/10.1002/aenm.201401882

[S14] K. Wang, S. Gao, Z. Du, A. Yuan, W. Lu, L. Chen, MnO_2-Carbon nanotube composite for high-areal-density supercapacitors with high rate performance. J. Power Sources **305**, 30–36 (2016). https://doi.org/10.1016/j.jpowsour.2015.11.064

[S15] S.-H. Ji, N.R. Chodankar, W.-S. Jang, D.-H. Kim, High mass loading of h-WO_3 and a-MnO_2 on flexible carbon cloth for high-energy aqueous asymmetric supercapacitor. Electrochim. Acta **299**, 245–252 (2019). https://doi.org/10.1021/acsami.8b09592

[S16] C. Xu, Z. Li, C. Yang, P. Zou, B. Xie et al., An ultralong, highly oriented nickel-nanowire-array electrode scaffold for high-performance compressible pseudocapacitors. Adv. Mater. **28**, 4105–4110 (2016). https://doi.org/10.1002/adma.201505644

[S17] T. Zhai, F. Wang, M. Yu, S. Xie, C. Liang et al., 3D MnO_2–graphene composites with large areal capacitance for high-performance asymmetric supercapacitors. Nanoscale **5**, 6790–6796 (2013). https://doi.org/10.1039/c3nr01589k

[S18] Z. Wang, F. Wang, Y. Li, J. Hu, Y. Lu, M. Xu, Interlinked multiphase Fe-doped MnO_2 nanostructures: A novel design for enhanced pseudocapacitive performance. Nanoscale **8**,

7309–7317 (2016). https://doi.org/10.1039/C5NR08857G

[S19] H. Wang, C. Xu, Y. Chen, Y. Wang, MnO_2 nanograsses on porous carbon cloth for flexible solid-state asymmetric supercapacitors with high energy density. Energy Storage Mater. **8**, 127–133 (2017). https://doi.org/10.1016/j.ensm.2017.05.007

[S20] Z.H. Huang, Y. Song, D.Y. Feng, Z. Sun, X. Sun, X.X. Liu, High mass loading MnO_2 with hierarchical nanostructures for supercapacitors. ACS Nano **12**, 3557–3567 (2018). https://doi.org/10.1021/acsnano.8b00621

[S21] J. Li, Y. Wang, W. Xu, Y. Wang, B. Zhang et al., Porous Fe_2O_3 nanospheres anchored on activated carbon cloth for high-performance symmetric supercapacitors. Nano Energy **57**, 379–387 (2019). https://doi.org/10.1016/j.nanoen.2018.12.061

[S22] R. Liu, Y. Wang, G. Yuan, Y. Liu, X. Li, Y. Bai, Y. Wang, High-performance flexible MnO_2@carbonized cotton textile electrodes for enlarged operating potential window symmetrical supercapacitors. Electrochim. Acta **299**, 12–18 (2019). https://doi.org/10.1016/j.electacta.2018.12.181

[S23] Y. Liu, X. Miao, J. Fang, X. Zhang, S. Chen et al., Layered-MnO_2 nanosheet grown on nitrogen-doped graphene template as a composite cathode for flexible solid-state asymmetric supercapacitor. ACS Appl. Mater. Interfaces **8**, 5251–5260 (2016). https://doi.org/10.1021/acsami.5b10649

[S24] Z. Pan, Y. Qiu, J. Yang, F. Ye, Y. Xu, X. Zhang, M. Liu, Y. Zhang, Ultra-endurance flexible all-solid-state asymmetric supercapacitors based on three-dimensionally coated MnO_x nanosheets on nanoporous current collectors. Nano Energy **26**, 610–619 (2016). https://doi.org/10.1016/j.nanoen.2016.05.053
